# Description of two new species of *Hisonotus* Eigenmann & Eigenmann, 1889 (Ostariophysi, Loricariidae) from the rio Paraná-Paraguay basin, Brazil

**DOI:** 10.3897/zookeys.395.6910

**Published:** 2014-04-01

**Authors:** Fábio F. Roxo, Cláudio H. Zawadzki, Waldo P. Troy

**Affiliations:** 1 Universidade Estadual Paulista, Departamento de Morfologia, Laboratório de Biologia e Genética de Peixes, Botucatu, São Paulo State, Brazil; 2 Universidade Estadual de Maringá, Departamento de Biologia, Núcleo de Pesquisas em Limnologia, Ictiologia e Aquicultura (Nupelia), Maringá, Paraná State, Brazil; 3 Universidade do Estadual de Mato Grosso, Departamento de Ciências Biológicas, Tangará da Serra, Mato Grosso State, Brazil

**Keywords:** Cascudinhos, fresh water, head plates, Hypoptopomatinae, Neotropical fish

## Abstract

Two new species of *Hisonotus* are described from the rio Paraná-Paraguay basin in Brazil. The most remarkable features of the new species are the odontodes forming longitudinally aligned rows (one odontode after the other, but not necessarily forming parallel series) on the head and trunk (*vs.* odontodes not forming longitudinally aligned rows), a pair of rostral plates at the tip of the snout (*vs.* a single rostral plate), the functional v-shaped spinelet (*vs.* spinelet non-functional, square-shaped, or absent). These features suggest close phylogenetic relationships with *Hisonotus bockmanni*, *H. insperatus*, *H. luteofrenatus* and *H. piracanjuba*. Additionally, both new species are distinguished from their congeners by characters related to head length and depth, orbital diameter, suborbital depth, caudal peduncle depth, pectoral-fin spine length, snout length and counts of teeth. *Hisonotus paresi* **sp. n.** further differs from its congeners by having contrasting dark geometric spots on the anterodorsal region of the body, a character lacking in *H. oliveirai* **sp. n.** The variation in number and shape of the rostral plate, posterior rostrum plates, infraorbitals and the preopercle in both new species and in *H. insperatus* are discussed.

## Introduction

Hypoptopomatinae is composed of 19 genera and about 135 valid species (Eschmeyer and Fong 2013). This group includes *Hisonotus* Eigenmann & Eigenmann, 1889, which has as type species *Hisonotus notatus* Eigenmann & Eigenmann, 1889. Regan (1904) considered *Hisonotus* to be a synonym of *Otocinclus* Cope, 1871. This taxonomy had been followed until Schaefer (1998a), who resurrected *Hisonotus* and listed the following combination of diagnostic characters: reduced or absent snout plates anterior to the nostril, the rostrum with enlarged odontodes, and thickened plates forming the lateral rostral margin. Additional characters used to distinguish *Hisonotus* from other genera of Hypoptopomatinae include a rostrum with enlarged odontodes and thickened plates forming the lateral rostral margins; the latter character is also present in some other species of Hypoptopomatinae, especially in species of *Microlepidogaster* Eigenmann & Eigenmann, 1889 (Britski and Garavello 2007).

The genus *Hisonotus* currently contains 31 valid species (Eschmeyer 2013), 16 of which described in the past decade. Two species from the upper rio Tapajós, *Hisonotus chromodontus* and *Hisonotus luteofrenatus*, were described by Britski and Garavello (2007). Later, four new species, *Hisonotus iota*, *Hisonotus leucophrys*, *Hisonotus megaloplax* and *Hisonotus montanus*, were described by Carvalho and Reis (2009) from the upper rio Uruguay. An examination of *Hisonotus* from the Laguna dos Patos system revealed an unexpectedly high local species richness of the genus including seven more new species – *Hisonotus armatus* Carvalho, Lehmann, Pereira & Reis, 2008, *Hisonotus brunneus* Carvalho & Reis, 2011, *Hisonotus carreiro* Carvalho & Reis, 2011, *Hisonotus heterogaster* Carvalho & Reis, 2011, *Hisonotus notopagos* Carvalho & Reis, 2011, *Hisonotus prata* Carvalho & Reis, 2011, and *Hisonotus vireo* Carvalho & Reis, 2011. Recently, three more new species were described – *Hisonotus piracanjuba* Martins & Langeani, 2012, *Hisonotus bockmanni* Carvalho & Datovo, 2012, and *Hisonotus bocaiuva* Roxo, Silva, Oliveira & Zawadzki, 2013. Herein, based on recent collection efforts, we add two new species to the genus *Hisonotus*: one from the upper rio Paraná basin and the other as the first species for this genus from the rio Paraguay basin.

## Material and methods

All measurements and counts were taken from the left side of the fish. Measurements were made from point to point to the nearest 0.1 mm with a digital caliper. Body plate and osteology nomenclature follows Schaefer (1997) and measurements follow Carvalho and Reis (2009) as shown in Table 1. Abbreviations used in the text followed Carvalho and Reis (2009). Morphometrics are given as percentages of standard length (SL), except for subunits of the head region that are expressed as percentages of head length (HL). Specimens were cleared and double stained (c&s) according to the method of Taylor and Van Dyke (1985). Vertebral counts also include the five vertebrae that comprise the Weberian apparatus. Dorsal-fin ray counts include the spinelet as the first unbranched ray. All examined specimens were collected according to the Brazilian laws, and are deposited under permanent scientific collection licenses. After collection, specimens were euthanized using 1% benzocaine in water, fixed in 10% formaldehyde and preserved in 70% alcohol. All samples are deposited at the DZSJRP, Departamento de Zoologia e Botânica, Universidade Estadual Paulista, São José do Rio Preto; LBP, Laboratório de Biologia e Genética de Peixes, Universidade Estadual Paulista, Botucatu; MCP, Museu de Ciências e Tecnologia, Pontifícia Universidade Católica do Rio Grande do Sul, Porto Alegre; MZUSP, Museu de Zoologia, Universidade de São Paulo, São Paulo; NUP, Coleção Ictiológica do Nupelia, Universidade Estadual de Maringá, Maringá; ZUEC, Museu de História Natural “Prof. Dr. Adão José Cardoso”, Universidade Estadual de Campinas, Campinas; ZMA, Zoologisches Museum, Universiteit van Amsterdam, Amsterdam. Zoological nomenclature follows the International Code of Zoological (4th Ed.).

**Table 1.** Morphometrics and meristics of *Hisonotus oliveirai* and *Hisonotus paresi*. SD = standard deviation.

	*Hisonotus oliveirai* n = 27				*Hisonotus paresi* n = 15			
	Holotype	Range	Mean	SD	Holotype	Range	Mean	SD
SL	26.4	22.8−28.4	24.4	1.43	26.2	18.0−26.2	22.7	2.99
**Percents of SL**								
Head length	36.5	35.6−41.1	37.7	1.41	39.2	36.1−41.7	39.4	1.44
Predorsal length	46.8	45.3−52.1	48.3	1.51	47.9	46.9−51.8	49.0	1.54
Dorsal-fin spine length	22.4	22.4−28.3	24.5	1.62	25.4	25.2−27.0	26.2	0.50
Anal-fin unbranched ray length	18.7	16.3−21.3	19.2	1.34	18.2	17.4−21.4	19.8	0.87
Pectoral-fin spine length	23.6	21.6−27.6	24.7	1.57	27.5	27.0−30.1	28.2	0.53
Pelvic-fin unbranched ray length	18.4	16.8−23.2	20.6	1.45	18.7	18.0−21.1	19.7	0.98
Cleithral width	24.6	23.8−26.8	25.3	0.89	23.5	22.2−24.3	23.3	0.49
Thoracic length	18.4	17.6−21.6	19.0	0.80	18.8	16.1−19.8	17.8	1.12
Abdominal length	21.9	17.9−22.3	20.5	1.24	21.5	16.2−21.6	19.0	1.82
Body depth at dorsal-fin origin	21.1	18.6−23.9	21.6	1.25	18.8	16.9−20.7	18.1	1.30
Caudal-peduncle length	28.3	26.3−31.5	29.3	1.18	27.5	25.3−29.8	27.7	1.61
Caudal-peduncle depth	10.5	10.8−12.5	11.4	0.64	10.6	10.2−11.3	10.7	0.27
**Percents of HL**								
Snout length	50.7	46.9−52.2	49.6	1.49	51.5	50.7−57.1	53.7	1.50
Orbital diameter	15.9	13.9−17.6	15.6	0.93	12.8	11.0−14.1	12.5	0.88
Interorbital width	35.2	32.1−37.1	34.9	1.52	32.8	32.4−36.0	34.2	1.21
Head depth	54.7	51.6−59.2	55.4	2.17	45.3	42.4−47.7	44.8	1.99
Suborbital depth	24.7	20.9−25.5	24.1	1.26	20.8	17.4−22.0	20.0	0.85
Mandibular ramus	11.2	6.8−12.9	10.7	1.12	6.0	6.0−8.0	6.8	0.57
**Meristics**	**Holotype**	**Low−High**	**Mode**	**SD**	**Holotype**	**Low−High**	**Mode**	**SD**
Left premaxillary teeth	13	11−18	14	2.0	10	6−10	8	1.37
Left dentary teeth	14	11−15	13	1.22	6	4−7	6	0.42
Left lateral scutes	24	24−25	24	0.64	24	24−25	24	0.48

## Results

### Hisonotus oliveirai sp. n.

http://zoobank.org/2D0CE389-F31D-48AE-8C62-E1C6531410DF

http://species-id.net/wiki/Hisonotus_oliveirai

Figure 1 ; Table 1

#### Holotype.

MZUSP 115061, 26.4 mm SL, female, Brazil, Paraná State, boundary between municipalities of Cambira and Apucarana, ribeirão Cambira, affluent of rio Ivaí, upper rio Paraná basin, 23°38'54"S, 51°29'58"W, coll. Zawadzki CH, de Paiva S, 29 October 2007.

**Figure 1.**
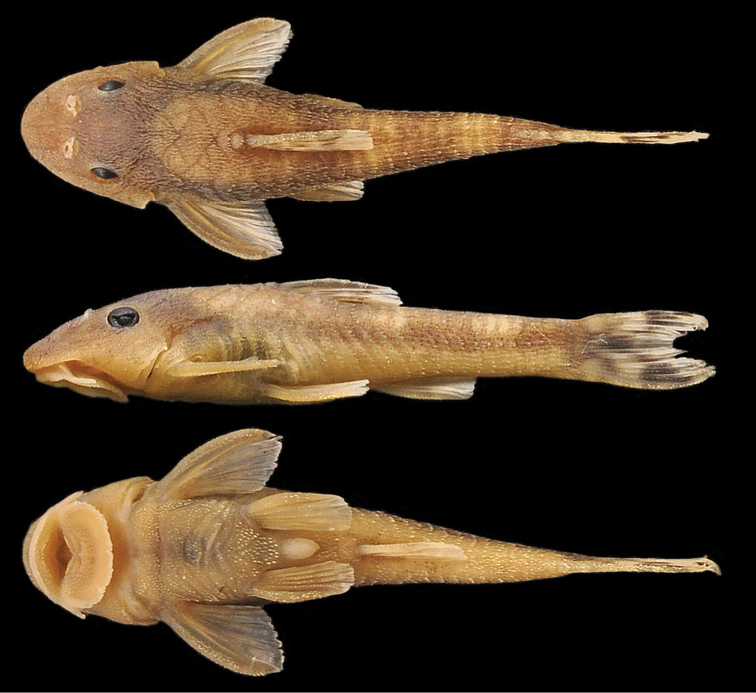
*Hisonotus oliveirai*, holotype, MZUSP 115061, female, 26.4 mm SL, from ribeirão Cambira, affluent rio Ivaí, upper rio Paraná basin, boundary between municipalities of Cambira and Apucarana, Paraná State, Brazil.

#### Paratypes.

All from Brazil, Paraná State. DZSJRP 18244, 3 males, 26.3−26.8 mm SL, ribeirão Salto Grande, rio Ivaí basin, municipality of Maria Helena, 23°37'08"S, 53°12'18"W, coll. Graça WJ, 30 December 2004. LBP 7358, 1 female, 28.4 mm SL, 1 unsexed, 12.4 mm SL, ribeirão Keller, rio Ivaí basin, boundary between municipalities of Marialva and Bom Sucesso, 23°38'30"S, 51°51'33"W, coll. Devidé R, 15 October, 2002. LBP 13332, 1 male, 23.2 mm SL, 1 unsexed c&s, 23.7 mm SL, rio Mourão, rio Ivaí basin, municipality of Campo Mourão, 24°02'23"S, 52°16'22"W, coll. Zawadzki CH, November 2010. LBP 13333, 1 male, 23.6 mm SL, 1 female, 25.4 mm SL, rio Mourão, rio Ivaí basin, municipality of Campo Mourão, 24°02'23"S, 52°16'22"W, coll. Pavanelli CS, 4 December 2006. LBP 13334, 1 male, 24.9 mm SL, ribeirão Keller, rio Ivaí basin, boundary between municipalities of Marialva and Bom Sucesso, 23°38'30"S, 51°51'32"W, coll. Zawadzki CH, November 2010. LBP 13335, 1 male, 26.0 mm SL, ribeirão Salto Grande, rio Ivaí basin, municipality of Maria Helena, 23°37'08"S, 53°12'18"W, coll. Graça WJ, 30 December 2004. LBP 14917, 4 females, 28.8−29.6 mm SL, 2 males, 26.6−27.4 mm SL, ribeirão Cambira, rio Ivaí basin, boundary between municipalities of Cambira and Apucarana, 23°58'54"S, 51°29'58"W, coll. Zawadzki CH, de Paiva S, 29 November 2007. LBP 17578, 3 females, 27.7−30.4 mm SL, 2 males, 25.4−26.1 mm SL, rio Mourão, rio Ivaí basin, boundary between municipalities of Engenheiro Beltrão and Quinta do Sol, 23°49'41"S, 52°11'43"W, coll. Zawadzki CH, Ruiz HB, Vieira RS, 01 April 2013. MCP 47860, 1 male, 25.6 mm SL, 1 female, 25.9 mm SL, ribeirão Salto Grande, rio Ivaí basin, municipality of Maria Helena, 23°37'08"S, 53°12'18"W, coll. Graça WJ, 30 December 2004. NUP 3578, 7 females, 27.8−28.1 mm SL, 8 males, 24.7−26.8 mm SL, 1 female c&s, 27.6 mm SL, 1 male c&s, 25.5 mm SL, ribeirão Salto Grande, rio Ivaí basin, municipality of Maria Helena, 23°37'08"S, 53°12'18"W, coll. Graça WJ, 30 December 2004. NUP 7065, 1 male, 23.3 mm SL, 1 female, 25.4 mm SL, 1 c&s unsexed, 24.5 mm SL, rio Mourão, rio Ivaí basin, municipality of Campo Mourão, 24°02'23"S, 52°16'22"W, coll. Zawadzki CH, 7 April 2009. NUP 9839, 1 male, 25.3 mm SL, 1 female, 25.8 mm SL, 1 female c&s, 25.0 mm SL, collected with holotype. NUP 15614, 10, 3 males, 25.9−26.5 mm SL, 7 females, 27.2−29.9 mm SL, rio Mourão, rio Ivaí basin, municipality of Engenheiro Beltrão, 23°37'41"S, 52°03'38"W, coll. Zawadzki CH, Ruiz HB, Silva HP, 22 October 2012. ZUEC 8006, 2, unsexed, 25.0−27.9 mm SL, rio Mourão, rio Ivaí basin, municipality of Engenheiro Beltrão, 23°37'41"S, 52°03'38"W, coll. Zawadzki CH, Ruiz HB, Silva HP, 22 October 2012. ZMA 250.056, 2, 1 male, 26.1 mm SL, 1 female, 25.6 mm SL, rio Mourão, rio Ivaí basin, municipality of Engenheiro Beltrão, 23°37'41"S, 52°03'38"W, coll. Zawadzki CH, Ruiz HB, Silva HP, 22 October 2012.

#### Diagnosis.

**Figure 2.**
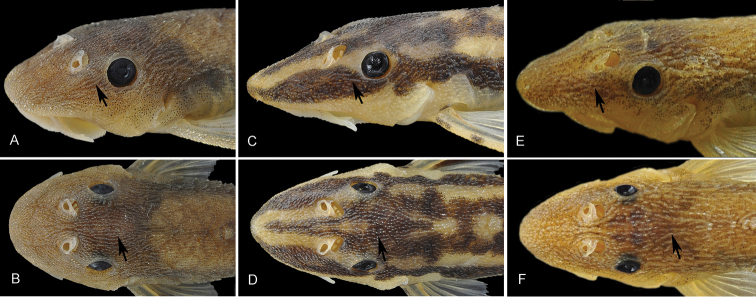
Variation in hypertrophied series of anterolateral (**A, C, E**) and anterodorsal (**B, D, F**) odontodes across three species. **A** *Hisonotus oliveirai*, paratype, NUP 9839, female, 25.8 mm SL, small odontodes **B** *Hisonotus oliveirai*, paratype, NUP 9839, female, 25.8 mm SL, small odontodes **C** *Hisonotus paresi*, paratype, NUP 10928, male, 24.2 mm SL, small odontodes **D** *Hisonotus paresi*, paratype, NUP 10928, male, 24.2 mm SL, small odontodes **E** *Hisonotus insperatus*, LBP 1316, 24.7 mm SL, large and conspicuous odontodes **F** *Hisonotus insperatus*, LBP 1316, 24.7 mm SL, large and conspicuous odontodes.

*Hisonotus oliveirai* can be distinguished from all congeners, except *Hisonotus insperatus* Britski & Garavello, 2003, *Hisonotus luteofrenatus* and *Hisonotus paresi*, by having odontodes forming longitudinally aligned rows (one odontode after the other, but not necessarily forming parallel series) on head and trunk, Fig. 2(A), (B) (*vs.* odontodes not forming longitudinally aligned rows). Additionally, the new species can be distinguished from all congeners except *Hisonotus insperatus*, *Hisonotus luteofrenatus*, *Hisonotus paresi*, and *Hisonotus piracanjuba* by having a pair of rostral plates at the tip of the snout (*vs.* a single rostral plate). Moreover, *Hisonotus oliveirai* can be further distinguished from all congeners except *Hisonotus bockmanni*, *Hisonotus chromodontus*, *Hisonotus insperatus*, *Hisonotus luteofrenatus*, and *Hisonotus paresi* by having a functional v-shaped spinelet (*vs.* spinelet non-functional, square-shaped, or absent). The new species can be distinguished from *Hisonotus bockmanni* and *Hisonotus paresi* by lacking contrasting dark geometric spots on the anterodorsal region of the body (*vs.* presence); from *Hisonotus insperatus* by having small, inconspicuous odontodes forming rows on the head and trunk (Fig. 2A, B; *vs.* large, conspicuous odontodes forming rows on the head and the trunk, Fig. 2E, F), a deeper head 51.6−59.2% HL (*vs.* 44.3−48.7% HL) and higher suborbital depth 20.9−25.5% HL (*vs.* 16.6−20.1% HL); from *Hisonotus luteofrenatus* by having a deeper caudal peduncle 10.8−12.5% SL (*vs.* 8.9−10.2% SL) and shorter snout 46.9−52.2% HL (*vs.* 67.0−75.3% HL); from *Hisonotus paresi* by a having deeper head 51.6−59.2% HL (*vs.* 42.4−47.7% HL), more premaxillary teeth 11−18 (*vs.* 6−10), and more dentary teeth 11−15 (*vs.* 4−7); from*Hisonotus piracanjuba* by having a deeper caudal peduncle 10.8−12.5% SL (*vs.* 8.3−9.5% SL), and shorter snout 46.9−52.2% HL (*vs.* 67.7−72.7% HL).

#### Description.

Morphometric data presented in Table 1. Maximum body length 28.4 mm SL. Dorsal profile of head slightly convex to straight from upper part of rostrum to posterior margin of nares, convex from eyes to posterior margin of parieto-supraoccipital, and straight to dorsal-fin origin. Dorsal proﬁle of trunk slightly concave and descending from dorsal-ﬁn origin to end of dorsal-fin base, straight to caudal peduncle. Ventral profile strongly concave from snout tip to opercular region; convex from opercular region to anal-fin origin; concave to caudal-fin insertion. Greatest body depth at dorsal-fin origin (18.6−23.9% SL). Greatest body width at opercular region, gradually decreasing towards snout and caudal fin. Cross-section of caudal peduncle almost ellipsoid; rounded laterally and almost flat dorsally and ventrally.

Head rounded in dorsal view, snout round to slightly pointed. Dorsal and ventral series of odontodes along anterior margin of snout completely covering its tip; odontodes larger than remaining ones on head. Odontodes on head and trunk hypertrophied and arranged in longitudinal rows (most prominent on head). Eyes moderately small (13.9−17.6% in HL), dorsolaterally positioned. Lips roundish with papillae uniformly distributed on base of dentary and premaxilla and slightly decreasing distally. Lower lip larger than upper lip; its border fringed. Maxillary barbel present; joined to lower lip by membrane for half its length. Teeth slender and bicuspid; mesial cusp larger than lateral cusp. Premaxillary teeth 11−18. Dentary teeth 11−15.

Dorsal-fin ii,7; dorsal-fin spinelet short and V-shaped; dorsal-fin lock functional; dorsal-fin origin slightly posterior to pelvic-fin origin. Tip of adpressed dorsal fin almost reaching end of anal-fin base. Pectoral-fin i,6; its tip almost reaching middle of pelvic-fin unbranched ray length when depressed. Pectoral axillary slit present between pectoral-fin insertion and lateral process of cleithrum. Pectoral spine supporting odontodes on ventral, anterior and dorsal surfaces. Pelvic-fin i,5; tip of pelvic-fin longest ray almost reaching anal-fin origin when depressed in females and reaching anal-fin origin in males. Pelvic-fin unbranched ray with dermal flap along its dorsal surface in males. Anal-fin i,5; its tip reaching 7th or 8th plate from its origin. Caudal-fin i,14,i; distal margin forked. Adipose-fin absent. Total vertebrae 27.

**Figure 3.**
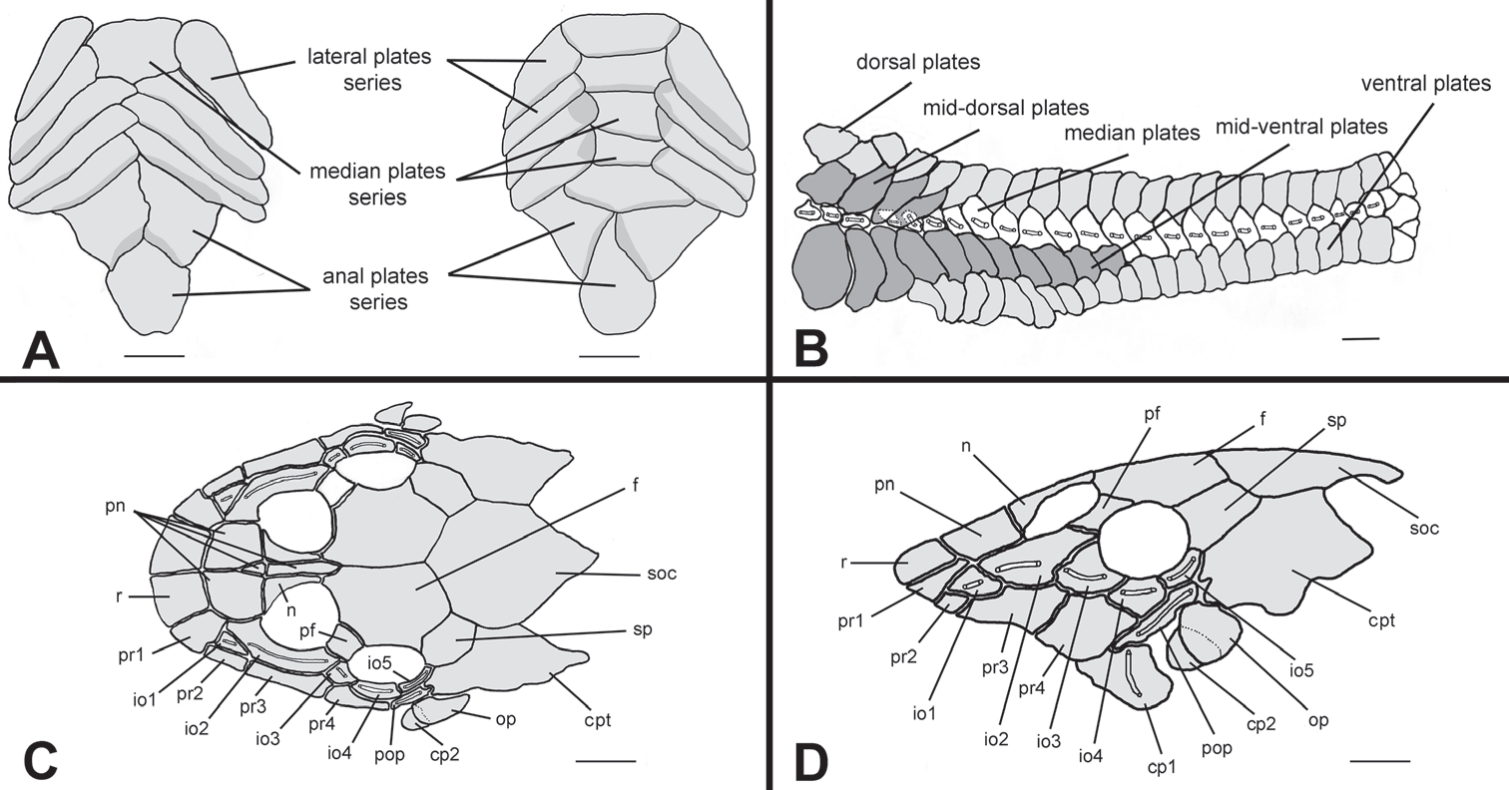
*Hisonotus oliveirai*, paratype, NUP 7065, sex unknown, 24.5 mm SL **A** Ventral view of abdominal region showing intraspecific variation in abdominal dermal plate patterns **B** lateral trunk plates; cranial bones and dermal plates of the head in dorsal **C** and lateral **D** view. Scale bars: 1 mm.

Body covered with bony plates except above lower lip, around pectoral and pelvic-fin origins and on dorsal-fin base. Cleithrum and coracoid totally exposed. *Arrector fossae* partially to completely enclosed by ventral lamina of coracoids. Abdomen entirely covered by plates (Fig. 3A); abdomen covered by large, elongate lateral plate series, formed by two lateral rows, approximately of same size; median plates formed by two patterns of plate distributions; first, median plate series not reaching anal shield plates with lateral plate series beginning to contact each other at middle of abdomen; second, median plate series reaching anal shield and lateral plate series remaining separate; anal plates series covered by large square or triangular plates. Body entirely covered laterally by plates (Fig. 3B); mid-dorsal plates poorly developed and reaching middle of dorsal-fin base; median plates series continuous in median portion of body; mid-ventral plates reaching vertical through end of dorsal-fin base.

Parts of dorsal head bone plates presented in Fig. 3C. Snout tip formed by one pair of square rostral plates (r). Nasal (n) rectangular, forming anterior medial nostril margin, posterior nasal margin contacting frontals (f), anterior and lateral margins contacting pre-nasals (pn). Pre-nasals (pn) positioned posterior to rostral plates (r); formed by two large square-shaped plates, one small and triangular and one elongated and rectangular between nares. Posterodorsal head plates consist of compound pterotic (cpt), parieto-supraoccipital (soc) and frontal (f; largest bones of head), prefrontal (pf) and sphenotic (sp). Compound pterotic (cpt) covered with few and small, unclustered fenestra. Lateral surface of head illustrated in Fig. 3D. Posterior rostrum plates pr1-pr2 smallest, rectangular shaped; pr4-pr3 largest, first rectangular and second square. Complete infraorbital plate series (io1-io5), present just above posterior rostrum series, all covered by laterosensory canal system; io2 largest and io5 smallest; io3, io4 and io5 forming inferior orbital margin of eyes. Preopercle (pop) elongate and rectangular, covered by laterosensory canal; preopercle present under pr4, io4 and io5, and upper cp1, cp2 and op. Subocular cheek plates (cp1-cp2) and opercle (op) form posterior lateral margin of head.

#### Coloration in alcohol.

Pale yellowish ground color. Dorsal surface of head dark brown, except for pale yellowish areas on snout tip, lateral margin of head and tip of parieto-supraoccipital. Three dark-brown saddles crossing dorsum, reaching longitudinal dark stripe on side of trunk: first below dorsal-fin origin, second typically at adipose-fin region, and third at end of caudal peduncle. Ventral region of anal-fin origin with small single-chromatophore spots. Caudal fin hyaline with two black bars; first at caudal-fin origin, second at middle of caudal fin (Fig. 1).

#### Sexual dimorphism.

**Figure 4.**
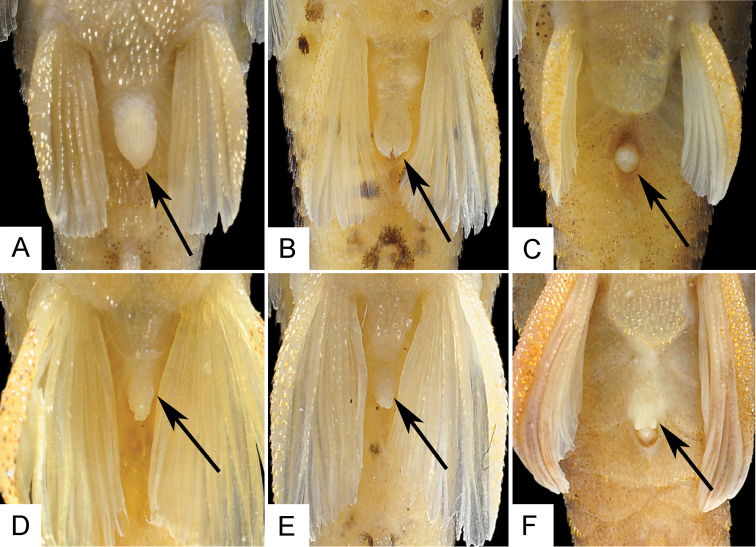
Ventral view of abdominal region of three species of *Hisonotus*, arrows indicate anal membrane in *Hisonotus oliveirai* (**A, D**) and *Hisonotus paresi* (**B, E**) contrasting with the lack of the anal membrane in *Hisonotus chromodontus* (**C, F**). **A** *Hisonotus oliveirai*, MZUSP 115061, holotype, female, 26.4 mm SL **B** *Hisonotus paresi*, MZUSP 115062, holotype, female, 26.2 mm SL **C** *Hisonotus chromodontus*, LBP 7964, female, 28.1 mm SL **D** *Hisonotus oliveirai*, NUP 3578, male, 27.1 mm SL **E** *Hisonotus paresi*, NUP 10928, male, 24.2 mm SL **F** *Hisonotus chromodontus*, LBP 12278, male, 26.7 mm SL.

Adult males are distinguished by having a papilla at the urogenital opening (*vs.* papilla absent in females); a pelvic ﬁn that extends beyond anal-ﬁn origin (*vs.* pelvic fin not reaching anal-fin origin in females); and an unbranched pectoral- and pelvic-fin ray supporting a dermal flap on their proximal dorsal surface in males. Both sexes have a membrane at anal opening; however, the membrane is longer and large in females (Fig. 4A) than in males (Fig. 4D), covering almost the entire urogenital opening.

#### Distribution.

**Figure 5.**
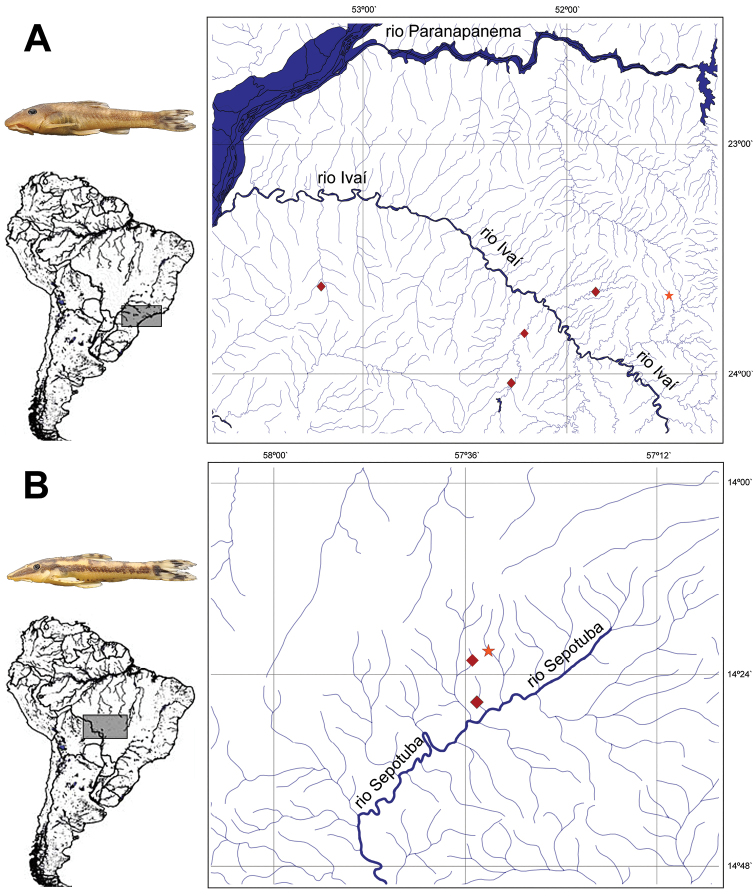
Map of the distribution of **A** *Hisonotus oliveirai*. Star = holotype locality, ribeirão Cambira. Diamonds = paratype localities **B** *Hisonotus paresi*. Star = holotype locality, riacho Águas Claras. Diamonds = paratype localities.

*Hisonotus oliveirai* is only known from four small to medium-sized streams, the ribeirão Salto Grande, ribeirão Keller, rio Mourão, and the ribeirão Cambira, all tributaries of the rio Ivaí in the upper rio Paraná basin (Fig. 5A).

#### Etymology.

The specific epithet *oliveirai* (a noun in the genitive case) is a patronym honoring professor Claudio Oliveira from the Universidade Estadual Paulista Júlio de Mesquita Filho (UNESP), Botucatu, São Paulo State, in recognition of his dedication and contributions to the studies of Neotropical freshwater fishes.

### Hisonotus paresi sp. n.

http://zoobank.org/FBC435D8-A305-4027-A3C5-5556971CFF8E

http://species-id.net/wiki/Hisonotus_paresi

Figure 6 ; Table 1

#### Holotype.

MZUSP 115062, 26.2 mm SL, female, Brazil, Mato Grosso State, municipality of Santo Afonso, riacho Águas Claras, affluent rio Sepotuba, rio Paraguay basin, 14°21'03"S, 57°33'07"W, coll. Troy WP, 14 September 2010.

**Figure 6.**
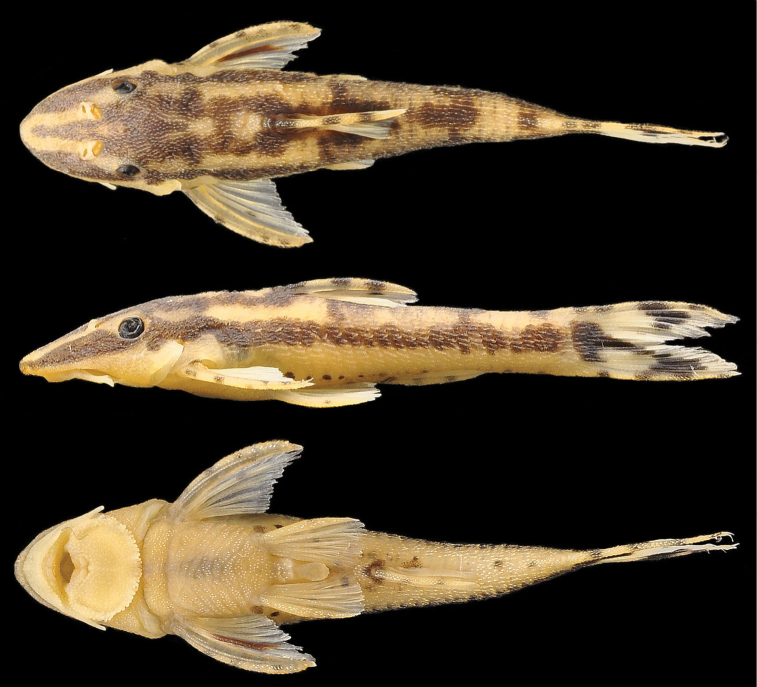
*Hisonotus paresi*, holotype, MZUSP 115062, female, 26.2 mm SL, riacho Águas Claras, affluent rio Sepotuba, rio Paraguay basin, municipality of Santo Afonso, Mato Grosso State.

#### Paratypes.

All from Brazil, Mato Grosso State, rio Sepotuba basin. DZSJRP 18245, 2 females, 19.9−24.3 mm SL, collected with holotype. LBP 13347, 2 females, 18.9−19.6 mm SL, collected with holotype. LBP 13351, 9, 14.7−24.3 mm SL, riacho Águas Claras, Santo Afonso, 14°21'03"S, 57°33'07"W, coll. Troy WP, April 2012. LBP 13352, 1, 23.7 mm SL, riacho Águas Claras, Santo Afonso, 14°21'03"S, 57°33'07"W, coll. Troy WP, April 2012. LBP 17532, 1 male 22.6 mm SL, 2 female 19.5−23.8 mm SL, 1 unsexed not measured, riacho Maracanã, boundary between municipalities of Santo Afonso and Nova Marilândia, 14°22'40"S, 57°35'11"W, coll. Troy WP, Paliga T, Silva VM, 3 April 2010. NUP 10928, 2 males, 23.2−24.2 mm SL, 2 c&s, 23.6−24.2 mm SL, 1 unsexed not measured, collected with holotype. NUP 10976, 3 unsexed, 16.7−20.5 mm SL, riacho São Jorge, municipality of Santo Afonso, 14°27'26"S, 57°34'34"W, coll. Zawadzki CH, Troy WP, 19 August 2010.

#### Diagnosis.

*Hisonotus paresi* can be distinguished from all congeners, except *Hisonotus bockmanni*, by the presence of contrasting dark geometric spots on the anterodorsal region of body (*vs.* absence of geometric spots). Additionally, the new species can be distinguished from all congeners, except *Hisonotus insperatus*, *Hisonotus luteofrenatus*, *Hisonotus oliveirai*, *Hisonotus piracanjuba*) by having a pair of rostral plates at the tip of the snout (*vs.* a single rostral plate). Also *Hisonotus paresi* can be distinguished from all congeners, except *Hisonotus insperatus*, *Hisonotus luteofrenatus* and *Hisonotus oliveirai* by having odontodes forming longitudinally aligned rows on head and trunk, Fig. 2C, D (*vs.* odontodes not forming longitudinally aligned rows). The new species can be distinguished from *Hisonotus bockmanni* by having a continuous median series of perforated plate (*vs.* median plate series of perforated plates discontinuous, that is, with a gap of unperforated plates), by lacking unpaired plates between the contra-lateral dorsal series (*vs.* having two tiny unpaired plates between the contra-lateral dorsal series, placed eight plates posterior to dorsal fin – see fig. 4 in Carvalho and Datovo 2012), and by having the anterior half of the caudal fin darkly pigmented medially (*vs.* caudal fin with anterior half hyaline); from *Hisonotus insperatus* by a longer pectoral-fin spine, 27.0−30.1% in SL (*vs.* 20.6−25.9%); from *Hisonotus luteofrenatus* by a longer head 36.1−41.7% SL (*vs.* 28.8−33.3%), smaller orbital diameter 11.0−14.1% HL (*vs.* 15.0−18.1%) and shorter snout 50.7−57.1% HL (*vs.* 67.0−75.3%); from *Hisonotus oliveirai* by lower head depth 42.4−47.7% HL (*vs.* 51.6−59.2%), fewer premaxillary teeth 6−10 (*vs.* 11−18) and fewer dentary teeth 4−7 (*vs.* 11−15); from *Hisonotus piracanjuba* by longer head 36.1−41.7% SL (*vs.* 27.9−32.2), deeper caudal peduncle 10.2−11.3% SL (*vs.* 8.3−9.5%), fewer premaxillary teeth 6−10 (*vs.* 14−22) and fewer dentary teeth 4−7 (*vs.* 12−19).

#### Description.

Morphometric data presented in Table 1. Maximum body length 26.2 mm SL. Lateral profile of head convex; straight from upper part of rostrum to posterior margin of nares, slightly curved from eyes to posterior margin of parieto supraoccipital, almost straight to dorsal-ﬁn origin. Dorsal proﬁle of trunk slightly concave, descending from base of dorsal-ﬁn origin to end of dorsal-fin base, straight to caudal peduncle. Ventral profile slightly concave from snout tip to pectoral-fin origin, convex to anal-fin origin, slightly concave to caudal peduncle. Greatest body depth at dorsal-fin origin (16.9−20.7% SL). Greatest body width at opercular region, gradually decreasing towards snout and caudal fin. Cross-section of caudal peduncle almost ellipsoid; rounded laterally and almost flat dorsally and ventrally.

Head rounded in dorsal view. Snout slightly pointed, its tip rounded, elongated (50.7−57.1% HL) and depressed in front of each nostril on dorsal surface. Dorsal and ventral series of odontodes completely covering anterior margin of snout; odontodes of snout similar in size to remaining ones found on head. Snout tip lacking band devoid of odontodes. Odontodes on head and trunk well deﬁned and arranged into longitudinal rows (character more prominent in head). Eyes small (11−14.1% HL), dorsolaterally positioned. Lips roundish and papillose; uniformly distributed on base of dentary and premaxilla and slightly decreasing distally. Lower lip larger than upper lip; its border strongly fringed. Maxillary barbel present. Teeth slender and bicupid; mesial cusp larger than lateral cusp. Premaxillary teeth 6−10. Dentary teeth 4−7.

Dorsal-fin ii,7; dorsal-fin spinelet short and V-shaped; dorsal-fin lock functional; its origin slightly anterior to pelvic-fin origin. Tip of adpressed dorsal-fin rays surpassing end of anal-fin base. Pectoral-fin i,6; tip of longest pectoral-fin ray almost reaching half of pelvic-fin length, when depressed. Pectoral axillary slit present between pectoral-fin insertion and lateral process of cleithrum. Pectoral spine supporting odontodes anteroventrally. Pelvic-fin i,5; its tip almost reaching anal-fin origin when depressed in females and reaching anal-fin origin in males. Pelvic-fin unbranched ray with dermal flap along its dorsal surface in males. Anal fin i,5; its tip reaching 7th and 8th from its origin. Caudal-fin i,14,i; distal margin emarginated. Adipose-fin absent. Total vertebrae 27.

**Figure 7.**
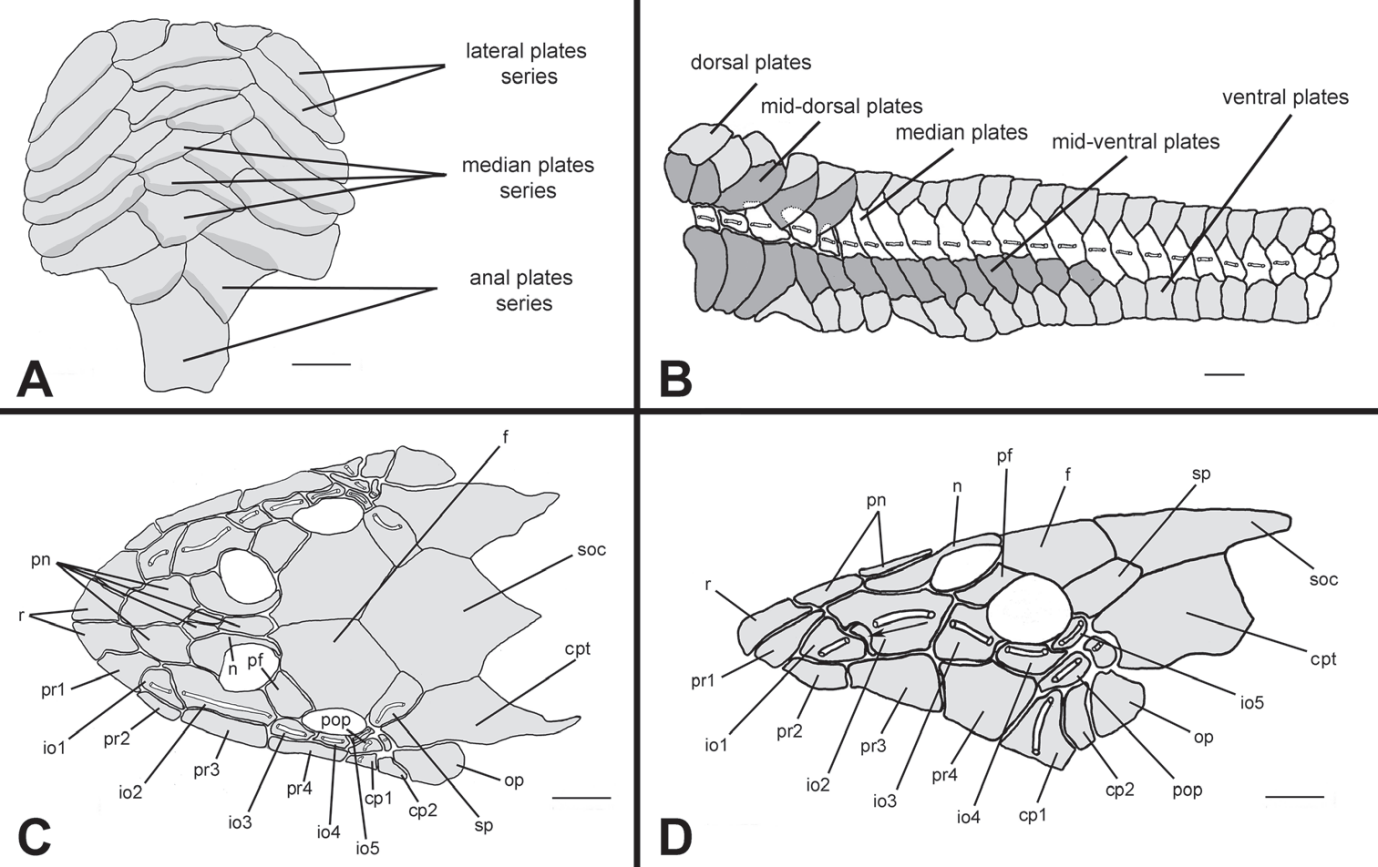
*Hisonotus paresi*, paratype, NUP 10928, male, 24.2 mm SL **A** Ventral view of abdominal plates **B** lateral trunk plates; cranial bones and dermal plates of the head in dorsal **C** and lateral **D** view. Black arrows (**D**) indicate an extra plate that is absent in the right side of the same specimen. Scale bars: 1 mm.

Body covered with bony plates except on ventral part of head, around pectoral and pelvic-fin origin and on dorsal-fin base. Cleithrum and coracoid totally exposed. *Arrector fossae* partially enclosed by ventral lamina of coracoids. Abdomen entirely covered by plates (Fig. 7A), abdomen formed by lateral plate series with elongate and large plates, formed by two lateral plates series, similar in size; median plates formed by one to three plates series reaching anal shield. Lateral of body entirely covered by plates (Fig. 7B); mid-dorsal plates poor developed, reaching middle of dorsal-fin base; median plates not interrupted in median portion of body; mid-ventral plates reaching end of dorsal-fin base.

Parts of dorsal head bone plates presented in Fig. 7C. Snout tip formed by one pair of rostral square-shaped plates (r). Nasal (n) almost rectangular forming anterior medial nostril margin in contact posteriorly with frontals (f) and anteriorly and laterally with pre-nasals (pn). Pre-nasals (pn) positioned posteriorly of rostral plates (r), formed by two large and one small square-shaped plates, and one elongate rectangular shaped between nares. Top of head composed by compound pterotic (cpt), parieto supraoccipital (soc) and frontal (f), largest bones of head, and prefrontal (pf) and sphenotic (sp). Compound pterotic (cpt) fenestrated randomly distributed. Lateral surface of head presented in Fig. 7D. Posterior rostrum plates pr1-pr2 small, and rectangular shaped; pr4-pr3 largest, first rectangular and second square-shaped. Infraorbital plate series complete (io1-io5), present just above posterior rostrum series, all covered by latero-sensory canal system; io2 largest and io5 smallest; io3, io4 and io5 forming inferior orbital margin of eyes; preopercle (pop) elongated and rectangular, covered by latero-sensory canal; preopercle present under io4 and io5, and upper cp1, cp2 and op. Subocular cheek plates (cp1-cp2) and opercle (op) form posterior lateral margin of head.

#### Coloration in alcohol.

Ground color of dorsal and ventral region of head and trunk pale yellowish. Conspicuous longitudinal dark stripe enlarging from rostral plates to anterior corner of eyes, straightening and bordering on ventral margin of eyes, enlarging again through compound pterotic and lateral series of plates to caudal-fin. Another conspicuous longitudinal dark stripe starting medially at pre-nasal plate region and enlarging on supraoccipital region. Unpigmented portion of snout appears as hyaline v-shaped mark from rostral plate passing through nares to orbital margins. Longitudinal dark stripe from superior portion of sphenotic through mid-dorsal plates to posterior margin of dorsal-fin base. Dark blotch on compound pterotic overlaps mid-dorsal longitudinal dark stripe. Dark saddle on middle portion of predorsal region reaches mid-dorsal longitudinal dark stripe. Overall, pigmentation pattern forms geometric spots on anterodorsal region of body. Three dark saddles usually cross posterodorsal region of body, reaching longitudinal stripe on side of trunk: first saddle at middle of dorsal fin, second at adipose-fin region, and third at end of caudal peduncle. Saddles inconspicuous in some specimens. Ventral region of body almost completely pale yellowish, except few dark spots on caudal peduncle and dark ring at anal-fin origin. Dorsal, pectoral, and pelvic fins with dark chromatophores forming irregular sets of bands: three on dorsal and pectoral fin, and one on pelvic fin. Anal fin with few scattered chromatophores, sometimes forming bands. Caudal fin hyaline, except for dark spot on origin of rays, and dark band on middle of rays (Fig. 6).

#### Sexual dimorphism.

Adults males have a papilla in urogenital opening (*vs.* absent in females); have a longer pelvic ﬁn that extends beyond anal-ﬁn origin (*vs.* pelvic fin not reaching anal-fin origin in females); and have an unbranched pelvic-fin ray supporting a dermal flap along its dorsal surface. Both sex have a membrane on the anal opening; however, this membrane is more developed in females (Fig. 4B) than in males (Fig. 4E), covering almost the entire urogenital opening.

#### Distribution.

The species is known from three small tributaries the riacho Águas Claras, riacho Maracaña and riacho São Jorge, all draining to the rio Sepotuba, in the upper rio Paraguay basin (Fig. 5B).

#### Etymology.

The species name *paresi* (a noun in apposition), refers to the the Paresí Indians who speak Paresí, a branch of the Aruak language. The Paresí used to live throughout most of Mato Grosso State including the municipality of Santo Afonso. Paresí were also some of the main guides of Marechal Cândido Rondon, the famous Brazilian pioneer in this region of Brazil at the beginning of the 18th century.

## Discussion

*Hisonotus oliveirai* is externally similar to *Hisonotus insperatus* and *Hisonotus piracanjuba* both species from upper stretches of the rio upper rio Paraná basin, *Hisonotus paresi* resembles more closely to *Hisonotus bockmanni* from the rio Tapajós basin. *Hisonotus insperatus*, *Hisonotus chromodontus*, *Hisonotus luteofrenatus*, and *Hisonotus oliveirai* have conspicuous odontodes forming well defined and widely spaced rows on the head and trunk (the main character used to distinguish theses species), while *Hisonotus paresi* has smaller, less conspicuous odontodes that form closely spaced rows (Fig. 2). Additionally, *Hisonotus insperatus*, *Hisonotus oliveirai* and *Hisonotus piracanjuba* have a deep head with a snout tip that rises abruptly to the interorbital region in lateral view, resulting in a short-snouted head profile. In *Hisonotus bockmanni*, *Hisonotus chromodontus*, *Hisonotus luteofrenatus* and *Hisonotus paresi*, the snout tip rises gently to the interorbital region in lateral view, resulting in a more long-snouted profile. The two snout patterns fit existing geographic patterns since *Hisonotus insperatus*, *Hisonotus oliveirai* and *Hisonotus piracanjuba* inhabit the upper rio Paraná while *Hisonotus paresi* is from the upper rio Paraguay and *Hisonotus bockmanni*, *Hisonotus chromodontus* and *Hisonotus luteofrenatus* are from the upper rio Tapajós. Such patterns among apparently closely related but now allopatric species suggest that the latter three species may have once shared a more broadly distributed ancestor. *Moenkhausia cosmops* Lima, Britski & Machado 2007, *Leporinus octomaculatus* Britski & Garavello, 1993, *Moenkhausia phaeonota* Fink, 1979, *Hyphessobrycon vilmae* Géry, 1966, and *Aequidens rondoni* Miranda-Ribeiro, 1918, *Parodon nasus* Kner, 1859, *Hemiodus semitaeniatus* Kner, 1858, are other examples of fishes occurring in the upper rio Paraguay basin, as well as in the upper rio Tapajós basin. Also, *Batrochoglanis melanurus* Shibatta & Pavanelli, 2005, which occurs at the upper rio Paraguay, appears to have its sister-taxon in the rio Tapajós basin. According to Hubert and Renno (2006) and Lima et al. (2007) these examples suggest that there may be a dispersal route between the upper rio Tapajós and the upper rio Paraguay basins.

Carvalho and Datovo (2012) reported a functional V-shaped spinelet as a character shared among *Hisonotus bockmanni*, *Hisonotus chromodontus*, *Hisonotus insperatus* and *Hisonotus luteofrenatus*, and this character is also present in *Hisonotus oliveirai* and *Hisonotus paresi*. They suggested that this is apparently synapomorphic within *Hisonotus*, and suggested that those species could compose a new monophyletic genus within the Hypoptopomatinae.

*Hisonotus paresi* has an unusual coloration pattern with contrasting dark stripes and bands converging to form geometric spots on the anterodorsal region of body, which is more similar in coloration to species of *Otocinclus* than to *Hisonotus*. However, *Hisonotus paresi* is morphologically similar to nominal species already assigned to *Hisonotus*, rather than to any other Hypoptopomatinae species. Additionally, *Hisonotus paresi* and *Hisonotus oliveirai* exhibit one of the diagnostic characters used to define *Hisonotus* in its resurrection by Schaefer (1998a): enlarged odontodes on rostrum. Thus, the aforementioned characters shared with *Hisonotus bockmanni*, *Hisonotus insperatus*, *Hisonotus luteofrenatus*, *Hisonotus oliveirai*, *Hisonotus paresi* and *Hisonotus piracanjuba* suggest a close phylogenetic relationship among these species.

**Figure 8.**
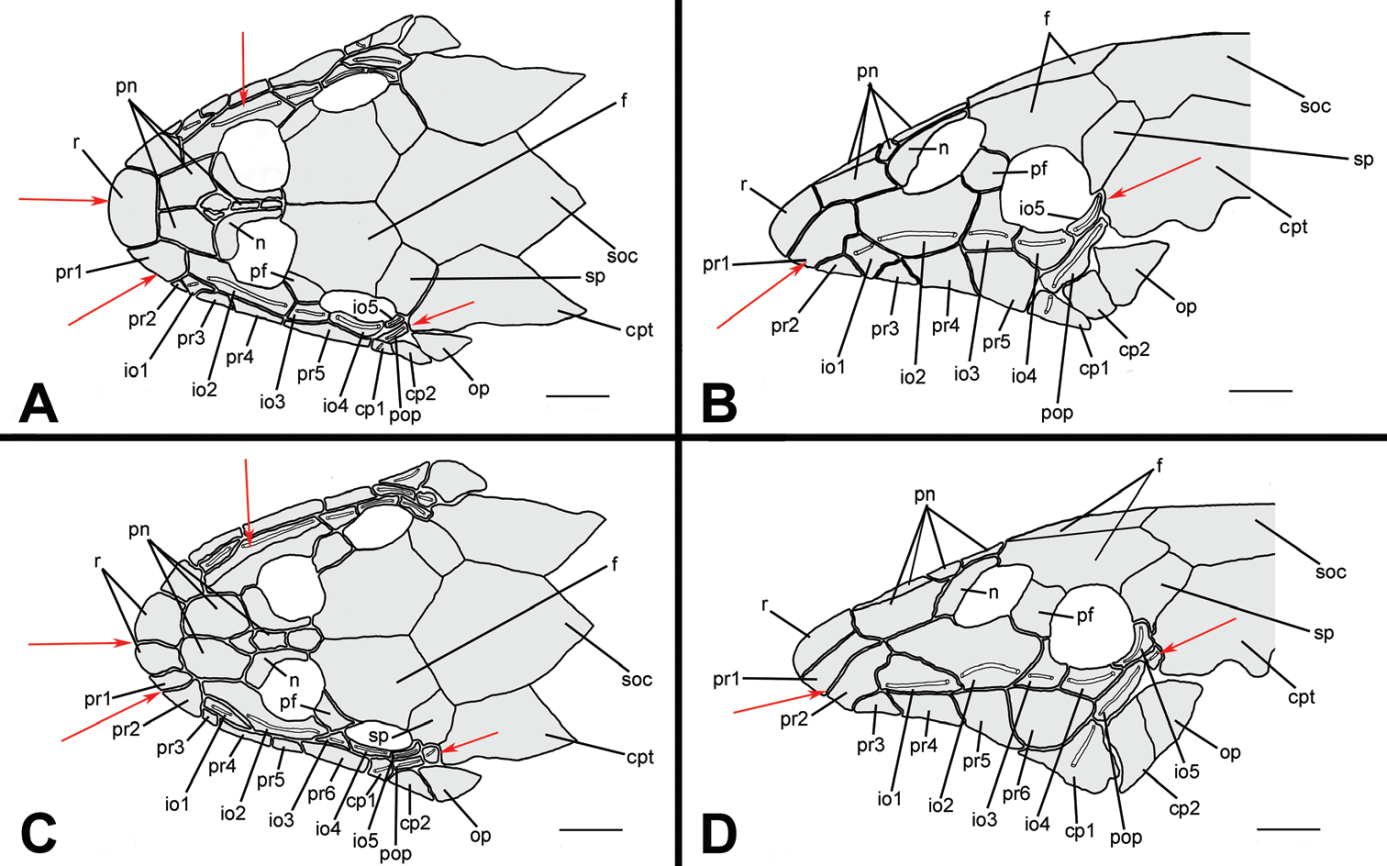
Cranial bones and dermal plates of the head of *Hisonotus insperatus* in dorsal (**A, C**) and lateral (**B, D**) view. Specimen illustrated in **A** and **B**: LBP 13336, female, 26.0 mm SL, from rio Capivara; specimen in **C** and **D**: LBP 13337, female, 28.6 mm SL, from rio Araquá, (both from Botucatu, São Paulo State). Red arrows indicate differences in osteology between the specimens. Scale bars: 1 mm.

Osteological characters are known to be conservative within Hypoptopomatinae species compared to external anatomy (Schaefer 1987, 1997, 1998b; Garavello 1977; Mo 1991; de Pinna 1998; Diogo et al. 2001; Ribeiro et al. 2005). Britski and Garavello (2003) used the presence of a pair of rostral plates in the snout tip to diagnose *Hisonotus insperatus*. Martins and Langeani (2012) also used that same character to distinguish *Hisonotus piracanjuba*. This character is present in both *Hisonotus oliveirai* and *Hisonotus paresi*. However, our results showed that the number and shape of head plates can be highly variable among specimens of a given species. We analyzed 18 cleared and stained specimens of *Hisonotus insperatus* from rio Capivara and rio Araquá from Botucatu, São Paulo State. Three individuals of *Hisonotus insperatus* had a single rostral plate, instead of a pair of rostral plates, however, all specimens of *Hisonotus oliveirai* and *Hisonotus paresi* had a pair of rostral plates. Variation in plate shape and number was further found in other head plates, including the posterior rostrum plates, infraorbitals and preopercle plate (red arrows in Fig. 8). For instance, the same specimen might have the fourth infraorbital divided in the right side, but not in the other left side Fig. 8C. This bilateral asymmetry was also found in a paratype of *Hisonotus oliveirai* (NUP 9839, 23.7 mm SL). Moreover, the first infraorbital of both sides might reach the ventral margin of the rostrum, among the second and third posterior rostrum plates (Fig. 8A, B), or not (Fig. 8C, D). Additionally, the size of the first infraorbital is variable among the specimens of *Hisonotus insperatus* and *Hisonotus oliveirai*. A similar pattern of variation was observed on posterior rostrum plates: the first and second posterior rostrum plates appear to be split only in the left side of the specimen (Fig. 8C, D), increasing the number of posterior rostrum plates to six, *versus* four in the right side. Finally, an extra plate is found among preopercle and compound pterotic perforated to infraorbital canal of the specimen of Fig. 8C, D, but not in the remaining specimens.

## Comparative material

All from Brazil, except when stated otherwise: *Hisonotus aky* Azpelicueta, Casciotta, Almirón & Koerber, 2004: MHNG 2643.039, 2, 33.1−34.2 mm SL, paratypes, arroio Fortaleza, Argentina; *Hisonotus bocaiuva* Roxo, Silva, Oliveira & Zawadzki, 2013: MZUSP 112204, male, 24.2 mm SL, holotype, córrego Cachoeira, Bocaiúva, Minas Gerais; LBP 9817, 9, 3 c&s, 18.3−23.2 mm SL, paratypes, córrego Cachoeira, Bocaiúva, Minas Gerais; *Hisonotus carreiro* Carvalho & Reis, 2011: MCP 40943, 3, 33.6−35.8 mm SL, arroio Guabiju, Guabiju, Rio Grande do Sul; *Hisonotus charrua* Almirón, Azpelicueta, Casciotta & Litz, 2006: LBP 4861, 1, 35.9 mm SL, arroio Guaviyú, Artigas, Uruguai; MHNG 2650.051, 1, 34.2 mm SL, paratype, arroio Aspinillar, Uruguay; *Hisonotus chromodontus* Britski & Garavello, 2007: LBP 7964, 25, 24.0−28.3 mm SL, 3 females c&s, 26.5−28.9 mm SL, 1 male c&s 24.9 mm SL, rio dos Patos, Nova Mutum, Mato Grosso; LBP 12278, 2, 26.7−28.7 mm SL, 1 unsexed c&s, 26.7 mm SL, rio Sumidouro, Tangará da Serra, Mato Grosso; MZUSP 45355, holotype, 25.9 mm SL, affluent rio Preto, Diamantino, Mato Grosso; *Hisonotus depressicauda* Miranda Ribeiro, 1918: MZUSP 5383, 24.4 mm SL, paralectotype (designated by Britski, 1969), Sorocaba; *Hisonotus francirochai* Ihering, 1928: LBP 13923, 22, 25.7−35.7 SL, córrego sem nome, Capitinga, Minas Gerais; MZUSP 3258, 29.4 mm SL, lectotype (designated by Britski 1969), rio Grande, São Paulo; *Hisonotus heterogaster* Carvalho & Reis, 2011: LBP 3335, 39, 20.8−30.1 mm SL, arroio sem nome, rio Grande, Rio Grande do Sul; *Hisonotus insperatus* Britski & Garavello, 2003: LBP 1299, 3, 23.5−29.6 mm SL, 1 female c&s, 24.8 mm SL, rio Araquá, Botucatu, São Paulo; LBP 1316, 2, 24.1−27.4 mm SL, 1 female c&s, 24.7 mm SL, 1 male c&s, 23.9 mm SL, rio Araquá, Botucatu, São Paulo; LBP 1344, 2, 22.9−24.9 mm SL, rio Araquá, Botucatu, São Paulo; LBP 1373, 1, 25.8 mm SL, rio Araquá, Botucatu, São Paulo; LBP 1405, 2, 22.2−27.3 mm SL, rio Araquá, Botucatu, São Paulo; LBP 4699, 17, 19.6−26.9 mm SL, 4 females c&s, 20.3−26.8 mm SL, 3 males c&s, 24.3−26.1 mm SL, ribeirão Cubatão, Marapoama, São Paulo; LBP 4945, 5, 27.3−28.5 mm SL, 2 females c&s, 28.2−29.9 mm SL, Botucatu, São Paulo; LBP 6770, 5, 25.1−28.2 mm SL, 3 females c&s, 20.0−27.0 mm SL, ribeirão Cubatão, Marapoama, São Paulo; LBP 13336, 1 female c&s, 26.0 mm SL, rio Capivara, Botucatu, São Paulo; LBP 13337, 2 females c&s, 27.4−28.6 mm SL, rio Araquá, Botucatu, São Paulo; MZUSP 22826, paratype, 1, 25.4 mm SL, córrego Água Tirada, Três Lagoas, Mato Grosso; MZUSP 24832, paratype, 1, 23.8 mm SL, rio Corumbataí, Corumbataí, São Paulo; MZUSP 78957, holotype, 29.6 mm SL, rio Capivara, Botucatu, São Paulo; MZUSP 78960, paratypes, 31, 12.6−26.0 mm SL, 5 c&s, 22.7−24.7 mm SL, rio Pardo, Botucatu, São Paulo; MZUSP 78965, paratypes, 10, 15.6−28.6 mm SL, 3 c&s, not measured, rio Araquá, Botucatu, São Paulo; MZUSP 78968, paratypes, 5, 24.1−27.3 mm SL, córrego da Figueira, Lins, São Paulo; *Hisonotus iota* Carvalho & Reis, 2009: LBP 13072, 5, 32.3−33.0 mm SL, rio Chapecó, Coronel Freitas, Santa Catarina; *Hisonotus laevior* Cope, 1894: LBP 3377, 1, 25.2 mm SL, arroio dos Corrientes, Pelotas, Rio Grande do Sul; LBP 6037, 8, 33.4−47.0 mm SL, rio Maquiné, Osório, Rio Grande do Sul; LBP 13187, 7, 19.4−45.8 mm SL, Córrego sem nome, Camaquá, Rio Grande do Sul; *Hisonotus leucofrenatus* Miranda Ribeiro, 1908: LBP 2085, 7, 38.3−50.6 mm SL, rio Sagrado, Morretes, Paraná; LBP 6837, 36, 35.1−43.5 mm SL, rio Fau, Miracatu, São Paulo; *Hisonotus leucophrys* Carvalho & Reis, 2009: LBP 13065, 6, 17.2−33.6 mm SL, rio Ariranhas, Xavantina, Santa Catarina; LBP 13073, 1, 36.8 mm SL, rio Guarita, Palmitinho, Rio Grande do Sul; *Hisonotus luteofrenatus* Britski & Garavello, 2007: MZUSP 62593, holotype, 28.6 mm SL, córrego Loanda, Cláudia, Mato Grosso; MZUSP 62594, paratype, 8, 22.4−30.5 mm SL, riacho Selma, Sinop, Mato Grosso; MZUSP 95940, 3, 26.1−28.5 mm SL, affluent rio Teles Pires, Itaúba, Mato Grosso; *Hisonotus maculipinnis* Regan, 1912: BMNH 1909.4.2.19−22, 1, 27.0 mm SL, syntype, rio de La Plata, Argentina; *Hisonotus megaloplax* Carvalho & Reis, 2009: LBP 13108, 6, 36.4−37.8 mm SL, Córrego sem nome, Saldanha Marinho, Rio Gande do Sul; *Hisonotus montanus* Carvalho & Reis, 2009: LBP 13051, 3, 26.4−27.2 mm SL, rio Goiabeiras, Vargem, Santa Catarina; LBP 13055, 5, 24.8−31.9 mm SL, rio Canoas, Vargem, Santa Catarina; *Hisonotus nigricauda* Boulenger, 1891: BMNH 1891.3.16.53−62, 1, 32.0 mm SL, syntype, Rio Grande do Sul; LBP579, 16, 34.1−40.1 mm SL, rio Guaíba, Eldorado do Sul, Rio Grande do Sul; *Hisonotus notatus* Eigenmann & Eigenmann, 1889: LBP 3472, 20, 21.0−34.3 mm SL, rio Aduelas, Macaé, Rio de Janeiro; LBP 10742, 25, 24.4−43.3 mm SL, rio Macabu, Conceição de Macabu, Rio de Janeiro; *Hisonotus paulinus* Regan, 1908: BMNH 1907.7.6.9, 28.4 mm SL, holotype, rio Piracicaba, São Paulo; *Hisonotus piracanjuba* Martins & Langeani, 2012: NUP 5059, 1, 24.7 mm SL, córrego Posse, Anápolis, Goiás; NUP 10979, 3, 21.4−21.8 mm SL, ribeirão Bocaina, Piracanjuba, Goiás; *Hisonotus prata* Carvalho & Reis, 2011: MCP 40492, 18, 19.5−33.2 mm SL, rio da Prata, Nova Prata, Rio Grande do Sul; LBP 9918, 14, 21.7−32.6 mm SL, Laguna dos Patos system, Nova Prata, Rio Grande do Sul; *Hisonotus ringueleti* Aquino, Schaefer & Miquelarena, 2001: FMNH 108806, 2, 25.7−32.2 mm SL, rio Quaraí basin, Uruguay; LBP 13148, 1, 24.5 mm SL, arroio Putiá, Uruguaiana, Rio Grande do Sul. *Microlepidogaster arachas* Martins, Calegari & Langeani, 2013: LBP 10882, 3, 22.8−35.3 mm SL, rio Paraná basin, Araxás, Minas Gerais; *Microlepidogaster dimorpha* Martins & Langeani, 2011: LBP 10683, 2, 28.8−35.6 mm SL; rio Paraná basin, Uberaba, Minas Gerais; *Otothyris travassosi* Garavello, Britski & Schaefer, 1998: LBP 1971, 13, 14.0−27.2 mm SL; coastal drainage, Canavieiras, Bahia; *Otothyropsis marapoama* Ribeiro, Carvalho & Melo, 2005: LBP 4698, 6, 23.9−36.3 mm SL; rio Tietê basin, Marapoama, São Paulo. *Parotocinclus* cf. *bahiensis* Miranda Ribeiro, 1918: LBP 7182, 3, 27.9−35.6 mm SL; rio Paraguaçu basin, Lençois, Bahia. *Parotocinclus maculicauda* Steindachner, 1877: LBP 2869, 15, 20.2−44.7 mm SL, rio Ribeira do Iguape basin, Miracatu, São Paulo; *Parotocinclus polyochrus* Schaefer, 1988: LBP 12272, 2, 21.2−22.6 mm SL, ribeirão Ínsula, Barra do Garça, Mato Grosso; *Parotocinclus prata* Ribeiro, Melo & Pereira, 2002: LIRP 1136, 38, 19.8−41.9 mm SL; rio São Francisco basin, Presidente Oligário, Minas Gerais.

## Supplementary Material

XML Treatment for Hisonotus oliveirai

XML Treatment for Hisonotus paresi
